# Enhanced thermal and photo-stability of a *para*-substituted dicumyl ketone intercalated in a layered double hydroxide

**DOI:** 10.3389/fchem.2022.1004586

**Published:** 2022-10-10

**Authors:** Ana L. Costa, Rodrigo P. Monteiro, Paulo D. Nunes Barradas, Simone C. R. Ferreira, Carla Cunha, Ana C. Gomes, Isabel S. Gonçalves, J. Sérgio Seixas de Melo, Martyn Pillinger

**Affiliations:** ^1^ Department of Chemistry, CICECO—Aveiro Institute of Materials, University of Aveiro, Aveiro, Portugal; ^2^ Coimbra Chemistry Centre (CQC)-IMS, Department of Chemistry, University of Coimbra, Coimbra, Portugal

**Keywords:** hexasubstituted ketones, ketodiacid, photodecarbonylation, intercalation, layered double hydroxides, myoglobin assay, CO-releasing molecules, TDDFT calculations

## Abstract

A ketodiacid, 4,4′-dicarboxylate-dicumyl ketone (**3**), has been intercalated into a Zn, Al layered double hydroxide (LDH) by a coprecipitation synthesis strategy. The structure and chemical composition of the resultant hybrid material (LDH-KDA3) were characterized by powder X-ray diffraction (PXRD), FT-IR, FT-Raman and solid-state ^13^C{^1^H} NMR spectroscopies, scanning electron microscopy (SEM), energy-dispersive X-ray spectroscopy (EDS), thermogravimetric analysis (TGA), and elemental analysis (CHN). PXRD showed that the dicarboxylate guest molecules assembled into a monolayer to give a basal spacing of 18.0 Å. TGA revealed that the organic guest starts to decompose at a significantly higher temperature (ca. 330°C) than that determined for the free ketodiacid (ca. 230°C). Photochemical experiments were performed to probe the photoreactivity of the ketoacid in the crystalline state, in solution, and as a guest embedded within the photochemically-inert LDH host. Irradiation of the bulk crystalline ketoacid results in photodecarbonylation and the exclusive formation of the radical-radical combination product. Solution studies employing the standard myoglobin (Mb) assay for quantification of released CO showed that the ketoacid behaved as a photoactivatable CO-releasing molecule for transfer of CO to heme proteins, although the photoreactivity was low. No photoinduced release of CO was found for the LDH system, indicating that molecular confinement enhanced the photo-stability of the hexasubstituted ketone. To better understand the behavior of **3** under irradiation, a more comprehensive study, involving excitation of this compound in DMSO-d_6_ followed by ^1^H NMR, UV-Vis and fluorescence spectroscopy, was undertaken and further rationalized with the help of time-dependent density functional theory (TDDFT) electronic quantum calculations. The photophysical study showed the formation of a less emissive compound (or compounds). New signals in the ^1^H NMR spectra were attributed to photoproducts obtained *via* Norrish type I α-cleavage decarbonylation and Norrish type II (followed by CH_3_ migration) pathways. TDDFT calculations predicted that the formation of a keto-enol system (*via* a CH_3_ migration step in the type II pathway) was highly favorable and consistent with the observed spectral data.

## 1 Introduction

The decarbonylation of carbonyl compounds such as aldehydes and ketones is an important transformation in synthetic chemistry with broad application in the synthesis of natural products (Akanksha and Maiti, 2012; Morgan et al., 2010; Natarajan et al., 2007; Ng et al., 2004) and the upgrading of biomass-derived molecules to bio-fuels and feedstock chemicals (Geilen et al., 2011; Huang et al., 2013; Chatterjee et al., 2018). In organic synthesis, selective decarbonylation reactions are typically mediated by a transition metal complex, either in a stoichiometric or catalytic fashion (Lu et al., 2021). Metal-free decarbonylation may be achieved photochemically for certain families of carbonyl compounds (Houk, 1976; Suzuki et al., 2014), or, more rarely, by thermolysis [e.g., of *R*-(+)-laurolenal to give *R*-(+)-1,2,3-trimethylcyclopentene (Crawford and Tokunaga, 1980)] or by “on water” reactions in the presence of molecular oxygen (as in the specific case of tertiary aldehydes) (Rodrigues et al., 2011). Besides its use in decarbonylative organic synthesis, decarbonylation has found widespread use in carbonylative transformations in which the carbonyl compound is the source of carbon monoxide (usually termed CO surrogate), thereby eliminating the need to supply CO externally from a gas cylinder (Wu et al., 2014; Cao et al., 2017; Konishi and Manabe, 2019). Examples of common CO surrogates are formic acid, formates, formaldehyde, formamides, *N*-formylsaccharin, and silacarboxylic acids. In a parallel line of research, metal-free carbonyl compounds are attracting interest as pharmaceutical agents for the controlled delivery of therapeutic amounts of CO to treat inflammatory diseases (Antony et al., 2013; Peng et al., 2013; Anderson et al., 2015; Abeyrathna et al., 2017; Ji and Wang, 2018; Slanina and Šebej, 2018; Soboleva and Berreau, 2019). These prodrugs are referred to as organic CO-releasing molecules (oCORMs) and many of them work by photoinduced decarbonylation.

The chemistry of UV or near-UV light-induced liberation of CO from small organic carbonyl compounds has been well studied since the early 1970s (Collins et al., 1970; Horspool and Khandelwal, 1970; Chapman and McIntosh, 1971; Mikol and Boyer, 1972). Selective formation of decarbonylated products is possible for certain combinations of carbonyl-containing substrates (with the right structural features) and reaction conditions (type of solvent in the case of solution-phase photolysis, presence or absence of oxygen, excitation wavelength). Examples of monodecarbonylation syntheses are α,β-unsaturated ketones from unsaturated lactones such as 2-(3*H*)-furanones (Chapman and McIntosh, 1971; Lohray et al., 1984; Gopidas et al., 1987), olefins from β,γ-unsaturated aldehydes (Baggiolini et al., 1970; Houk, 1976), alkynes from cyclopropenones (Poloukhtine and Popik, 2003), and various compounds from furan-2,3-diones [e.g., salicylic acid derivatives from coumarandiones in the presence of certain nucleophiles (Horspool and Khandelwal, 1970)]. A well-studied photobisdecarbonylation is the conversion of α-diketones to acenes (Suzuki et al., 2014). These earlier studies laid the foundations for the recent and ongoing development of photoactivatable CORMs, which now encompass cyclic xanthene-9-carboxylic acid (Antony et al., 2013), α-diketones (Peng et al., 2013), 3-hydroxyflavones (Anderson et al., 2015; Soboleva and Berreau, 2019), 3-hydroxy-4-oxoquinolines (Soboleva and Berreau, 2019), and diphenyl cyclopropenone-centered polymers (Shao et al., 2020), among others. Another highly productive and innovative branch of photodecarbonylation chemistry is the study of reactions in the solid state. Garcia-Garibay and co-workers have shown that the photoinduced decarbonylation of crystalline hexasubstituted ketones can cleanly and quantitatively afford compounds with adjacent quaternary stereogenic centers in one step (Mortko and Garcia-Garibay, 2005; Veerman et al., 2006; Family and Garcia-Garibay, 2009; Shiraki et al., 2011). In contrast, reactions in solution tend to yield mixtures of disproportionation and combination products. The high selectivity and specificity of the photodecarbonylation reactions in the crystalline state is due to confinement effects together with the geometrical constraints imposed by the rigid crystal lattice.

To the best of our knowledge, the photodecarbonylation of hexasubstituted ketones in the solid-state has only been studied for close-packed organic crystals. Supramolecular chemistry provides, however, several other strategies to control photoreactions through confinement of a substrate within an organizing medium, better known as host-guest chemistry (Ramamurthy and Gupta, 2015; Ramamurthy and Sivaguru, 2016). Examples of hosts which have been investigated include inorganic porous and layered materials [zeolites, clays, layered double hydroxides (LDHs)] (Ogawa and Kuroda, 1995; Scaiano and García, 1999; Shichi and Takagi, 2000; Ramamurthy, 2019), metallo-cages (Pd nanocage) (Karthikeyan and Ramamurthy, 2007; Ramamurthy and Gupta, 2015; Ramamurthy and Sivaguru, 2016), and organic molecular containers (cyclodextrins, cucurbiturils, calixarenes, octa-acid cavitand) (Karthikeyan and Ramamurthy, 2007; Ramamurthy, 2015; Ramamurthy and Gupta, 2015; Ramamurthy and Sivaguru, 2016; Pattabiraman et al., 2018). Among inorganic hosts, the interlayer region of LDHs provides an expandable two-dimensional (2D) reaction field for spatially controlled photochemical transformations (Ogawa and Kuroda, 1995; Newman and Jones, 1998). A range of organic guests may be incorporated (Newman and Jones, 1998). Normally, the geometrical constraint imposed by the host LDH layers leads to a highly ordered and well-defined arrangement of the guests inside the interlayer region. The main distinguishing feature of LDHs is that the guest species must possess a net negative charge to counterbalance the positive charge of the mixed metal hydroxide layers.

Among the hexasubstituted ketones studied by Garibay and co-workers, several of the photoreactive substrates were functionalized with carboxylate groups (Mortko and Garcia-Garibay, 2005; Family and Garcia-Garibay, 2009). These molecules should be ideal guests for LDHs. We therefore decided to prepare supramolecular LDH assemblies containing these ketoacids which would provide an opportunity to study the solid-state photodecarbonylation properties in a 2D reaction field. Here we report the synthesis and characterization of a zinc-aluminium LDH intercalated by a di-*p*-dicarboxylic acid derivative of dicumyl ketone, and a comparison of the photochemistry (in terms of photoproduct selectivities) of the ketodiacid in the crystalline state, in solution, and as a guest embedded within the LDH host.

## 2 Experimental

### 2.1 Materials and methods

The chemicals *N*,*N*′-dicyclohexylcarbodiimide, 4-(dimethylamino)pyridine, potassium hydride (30% in mineral oil), methyl iodide, periodic acid, chromium(VI) oxide, Zn(NO_3_)_2_·6H_2_O (98%, Fluka), Al(NO_3_)_3_·9H_2_O (98.5%, Riedel de-Haën), 1 M NaOH (Fluka), Na_2_S_2_O_4_ (Panreac), phosphate buffered saline (PBS) tablet, and 4-(2-hydroxyethyl)piperazine-1-ethanesulfonic acid (HEPES, 99.5%) were obtained from commercial sources (Sigma-Aldrich, unless otherwise indicated) and used as received. Lyophilized horse heart myoglobin and PBS solution were acquired from Sigma-Aldrich. THF (Aldrich) was dried over 4 Å molecular sieves. A nitrate-form Zn-Al LDH (denoted LDH-NO_3_) with the composition Zn_4_Al_2_(OH)_12_(NO_3_)_2_·2.5H_2_O was prepared by using the standard method of coprecipitation of the Zn^2+^ and Al^3+^ hydroxides (initial Zn^2+^/Al^3+^ molar ratio in solution = 2) in the presence of nitrate ions at a constant pH of 7.5–8 under nitrogen, followed by aging of the gel at 80°C for 20 h (Gomes et al., 2013; Costa et al., 2017).

FT-IR spectra were collected using KBr pellets and a Mattson-7000 infrared spectrophotometer. Solution ^1^H NMR spectra were recorded either on a Bruker Avance III spectrometer operating at 400.13 MHz (for irradiated DMSO-d_6_ solutions) or (for all other measurements) a Bruker-AMX spectrometer. Solution ^13^C{^1^H} NMR spectra were recorded on a Bruker-AMX spectrometer with an operating frequency of 101 MHz. Solid-state ^13^C{^1^H} cross-polarization (CP) magic-angle spinning (MAS) NMR spectra were recorded using a wide-bore Bruker Avance III 400 spectrometer (9.4 T) at 100.62 MHz with 3.7 µs ^1^H 90° pulses, 3.5 ms contact time, spinning rates of 12 kHz, and 5 s recycle delays.

Microanalyses for C, H, and N were carried out with a Truspec Micro CHNS 630-200-200 elemental analyzer. Powder X-ray diffraction (PXRD) data were collected at ambient temperature on a Philips Analytical Empyrean diffractometer equipped with a PIXcel 1D detector, with automatic data acquisition (X’Pert Data Collector software version 4.2) using monochromatized Cu-K_α_ radiation (*λ* = 1.54178 Å). Intensity data were collected by the step-counting method (step 0.02°), in continuous mode, in the 2*θ* range 3–70°. Scanning electron microscopy (SEM) images were obtained on a Hitachi SU-70 microscope at 15 kV. Samples were prepared by deposition on aluminium sample holders followed by carbon coating using an Emitech K 950 carbon evaporator. Thermogravimetric analysis (TGA) was performed using a Hitachi STA300 system at a heating rate of 5°C min^−1^ under air.

Baseline-corrected absorption spectra for the Mb assays were measured from 200 to 800 nm at a scanning rate of 600 nm/min on a Cary 5000 UV-Vis-NIR spectrometer. Alternatively, a Shimadzu 2600 was used to obtain the UV-Vis absorption spectra, and a Horiba-Jobin-Yvon Spex Fluorolog 3-2.2. spectrophotometer, corrected for the instrumental response of the system, was used to record the fluorescence spectra.

Structural models and representations were generated using CrystalMaker software (CrystalMaker Software, 2017).

### 2.2 Synthesis

#### 2.2.1 1,3-Di-*p*-tolylpropan-2-one (1)

**SCHEME 1:**
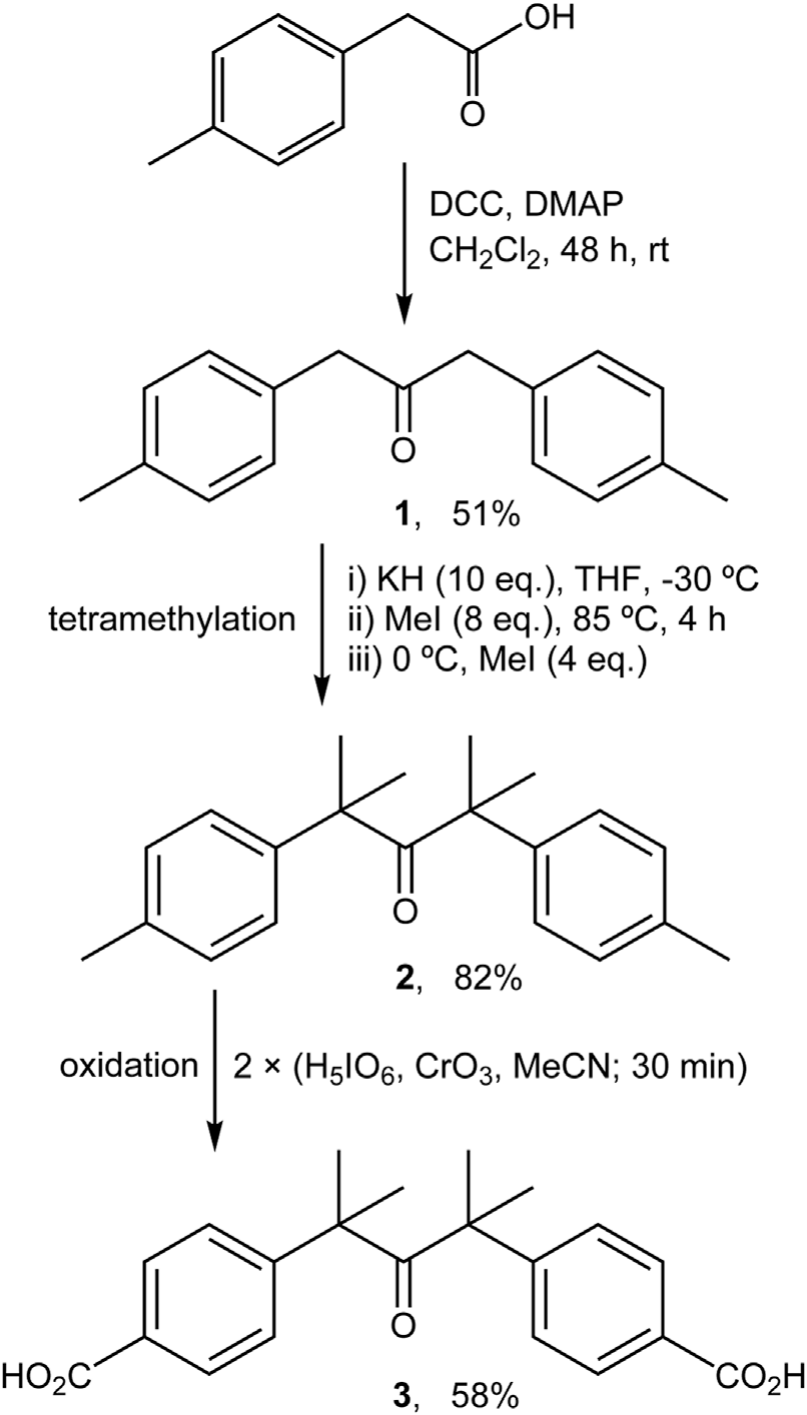
Preparation of ketodiacid **3** *via* modified literature procedures (Resendiz and Garcia-Garibay, 2005; Family and Garcia-Garibay, 2009).

The procedure described by Resendiz and Garcia-Garibay (2005) was followed with slight modifications (Scheme 1). A Schlenk tube was charged with dicyclohexylcarbodiimide (6.23 g, 30.2 mmol) and 4-(dimethylamino)pyridine (0.93 g, 7.61 mmol) in CH_2_Cl_2_ (45 ml) under inert atmosphere. A solution of *p*-tolylacetic acid (4.50 g, 30.0 mmol) in CH_2_Cl_2_ (40 ml) was then added dropwise with stirring. The resultant yellow-orange mixture was stirred for 48 h at room temperature, and then the precipitated solid was removed by filtration, washed with CH_2_Cl_2_, and volatiles were removed from the filtrate by evaporation under reduced pressure. Purification of the residue by chromatography (hexane/dichloromethane 97:3) gave the substituted dibenzyl ketone **1** (1.84 g, 51%). ^1^H NMR (400 MHz, DMSO-d_6_): *δ* = 2.27 (s, 6H, CH_3_), 3.75 (s, 4H, CH_2_), 7.03 (m, 4H, aryl-CH), 7.11 (m, 4H, aryl-CH).

#### 2.2.2 2,4-Dimethyl-2,4-di-*p*-tolylpentan-3-one (2)

The procedure described by Resendiz and Garcia-Garibay (2005) was followed with slight modifications (Scheme 1). A solution of compound **1** (0.80 g, 3.36 mmol) in dry THF (30 ml) was added dropwise to a magnetically stirred and cooled (−30°C; acetone/liq. N_2_ bath) suspension of potassium hydride (1.35 g, 33.6 mmol) in dry THF (50 ml). After dropwise addition of methyl iodide (1.68 ml, 26.8 mmol), the yellow suspension turned pale yellow, and was heated to 85°C and refluxed for 4 h, during which time a further color change to orange occurred. The mixture was cooled to 0°C in an ice bath and an additional amount of methyl iodide (0.84 ml, 13.4 mmol) was added. After evaporation of the solvent under reduced pressure, the residue was washed with water and extracted with diethyl ether. Concentration of the organic phase yielded the derivative **2** as a pale yellow powder (0.81 g, 82%). ^1^H NMR (400 MHz, DMSO-d_6_): *δ* = 1.17 (s, 12H, CH_3_), 2.28 (s, 6H, CH_3_), 7.06 (m, 4H, aryl-CH), 7.12 (m, 4H, aryl-CH).

#### 2.2.3 4,4′-(2,4-dimethyl-3-oxopentane-2,4-diyl)dibenzoic acid (3)

The procedure described by Family and Garcia-Garibay (2009) was followed with significant modifications (Scheme 1). A solution of periodic acid (2.31 g, 10.1 mmol) in acetonitrile (80 ml) was prepared with vigorous magnetic stirring. Then, chromium(VI) oxide (0.08 g, 0.75 mmol) was added and the color of the solution changed immediately to orange. Finally, compound **2** (0.57 g, 1.94 mmol) was added and the resultant yellow suspension was stirred for 30 min. At this point a second addition of a freshly prepared H_5_IO_6_/CrO_3_/MeCN mixture (in amounts equal to those used initially) was made and the mixture was stirred for a further 30 min. The solvent was then removed under reduced pressure and the resultant solid was washed several times with Milli-Q water. Purification of the product by recrystallization from acetone:water (20 ml: 40 ml) gave the ketodiacid **3** as a white powder (0.40 g, 58%). ^1^H NMR (400 MHz, DMSO-d_6_): *δ* = 1.25 (s, 12H, CH_3_), 7.28 (d, 4H, aryl-CH, *J* = 8.4 Hz), 7.85 (d, 4H, aryl-CH, *J* = 8.4 Hz). ^13^C NMR (101 MHz, DMSO-d_6_): *δ* = 27.5, 53.0, 126.0, 129.0, 129.5, 148.9, 167.1, 211.7.

#### 2.2.4 Layered double hydroxide-KDA3

A solution of the sodium salt of the diacid **3** was prepared by dissolving **3** (0.66 g, 1.86 mmol) in decarbonated deionized (DD) water (30 ml) along with 2 equiv. of 0.25 M NaOH. A solution of Zn(NO_3_)_2_·6H_2_O (1.11 g, 3.74 mmol) and Al(NO_3_)_3_·9H_2_O (0.70 g, 1.87 mmol) in DD water (30 ml) was added dropwise to the above solution, under nitrogen atmosphere, and 0.25 M NaOH was added simultaneously to maintain the pH of the reaction mixture at 8. Once addition of the Zn^2+^/Al^3+^ solution was complete, the resultant white suspension was stirred for 18 h at 65°C (final pH = 8.4). The solid product was recovered by filtration, washed several times with DD water (0.5 L), and dried at room temperature under reduced pressure in a vacuum desiccator. Anal. Calcd for Zn_4.3_Al_2_(OH)_12.6_(C_21_H_20_O_5_)_0.92_(NO_3_)_0.16_(H_2_O)_5_ (973.62): C, 23.83; H, 4.24; N, 0.23. Found: C, 23.84; H, 4.24; N, 0.23%. EDS gave an average Zn/Al atomic ratio of 2.15 ± 0.05. TGA revealed a mass loss of 8.9% from ambient temperature up to 150°C (calcd for 5H_2_O: 9.2%).

### 2.3 Photolysis experiments

Photolysis experiments were carried out using a 158.5 W medium pressure Peschl Ultraviolet mercury arc lamp (catalog No. 50043) equipped with a circulating water cooled pyrex jacket. Supplementary Figure S1 shows a typical setup for a myoglobin (Mb) UV-Vis assay in which the cuvette containing the test solution is placed at a distance of 5 cm from the lamp. A similar arrangement was used to irradiate solid samples or DMSO-d_6_ solutions of **3** in an NMR tube.

### 2.4 Deintercalation tests

The stability of LDH-KDA3 in different aqueous media was explored through the following experiments: 1) A sample of LDH-KDA3 (27 mg) was irradiated in the solid-state for 12 h. The solid (iLDH) was then added to a solution of Na_2_CO_3_ (125 mg, 1.18 mmol) in deionized water (7 ml), and the suspension was stirred overnight at rt. The resultant solid (designated iLDH^DI^, where DI stands for deintercalated) was recovered by filtration, washed with deionized water (2 × 5 ml), and vacuum-dried at rt. A second solid designated as iKDA^DI^ was recovered from the filtrate by evaporation of the solution to dryness. This procedure was also performed for non-irradiated LDH-KDA3, giving the solids LDH^DI^ and KDA^DI^. 2) In two parallel experiments, LDH-KDA3 (20 mg) was incubated in 0.01 M PBS or 0.01 M HEPES buffer solutions at rt for 5 h. The resultant solids, designated as LDH^PBS^ and LDH^HEPES^, were recovered by filtration, washed with deionized water (2 × 10 ml), and vacuum-dried at rt.

### 2.5 Myoglobin assay

The photoinduced release of CO from the synthesized compounds was assessed by using the Mb assay, in which the conversion of deoxymyoglobin (deoxy-Mb) to carbonmonoxy-myoglobin (MbCO) can be spectrophotometrically measured (Motterlini et al., 2002).

The heme group functions as the active site of Mb, originating two π → π* electronic transitions: one, very intense, at about 400 nm (Soret or B band), and a second at 500–600 nm (the Q bands). The wavelengths corresponding to these wavelength maxima are governed by the oxidation, spin, and coordination states of the heme iron. As a result, it is possible to distinguish between different forms of Mb by the respective peak positions and relative optical density values of the absorption spectra.

All experiments were performed by using a stock solution of Mb, which was freshly prepared by dissolving the protein in degassed (O_2_ free) 0.1 M PBS (pH = 7.4). An aliquot of this solution was taken and bubbled with gaseous nitrogen (99.99% pure), to which freshly prepared 1 M Na_2_S_2_O_4_ (300 μl) was added to promote conversion of met-myoglobin into deoxy-Mb. While the solution was being bubbled, a solution (2 ml) of the ketone was added and the solution made up to 3 ml with 0.1 M PBS. After 15 min of bubbling, this solution was stored in quartz cuvettes with a magnetic stirring bar, with an optical path of 1 cm, and sealed with a Teflon stopper and parafilm to prevent any escape or entry of gas. An absorbance spectrum was immediately acquired after preparation of the samples. These were subsequently irradiated at room temperature and absorption spectra of the irradiated solutions were recorded periodically.

Blank solutions of deoxy-Mb with the same concentration for each experiment were prepared simultaneously and their absorption spectra were measured; these blank solutions were immediately saturated with CO gas at 10 bar for at least 1 h to promote a complete conversion of deoxy-Mb to MbCO using an autoclave reactor.

The CO released was quantified by following the absorbance of samples at 540 nm as reported previously (Atkin et al., 2011), using blank solutions and four isosbestic points between deoxy-Mb and MbCO in Q Bands to normalize data.

### 2.6 Time-dependent density functional theory studies

All theoretical calculations were of the DFT type, carried out using version R3 of GAMESS-US (Schmidt et al., 1993). A range-corrected LC-BPBE ( ω=0.20 au^−1^) functional, as implemented in GAMESS-US (Schmidt et al., 1993), was used in both ground- and excited-state calculations. TDDFT calculations, with similar functionals, were used to probe the excited-state potential energy surface (PES). A solvent was included using the polarizable continuum model with the solvation model density to add corrections for cavitation, dispersion, and solvent structure. In TDDFT calculation of FC (Franck-Condon) excitations the dielectric constant of the solvent was split into a “bulk” component and a fast component, which is essentially the square of the refractive index. In “adiabatic” conditions only the static dielectric constant is used. A 6-31G** basis set was used in either DFT or TDDFT calculations. The results obtained with the LC-BPBE(20) functional are essentially unscaled raw data from calculations; for the S_0_→S_n_ transitions, a small correction, which results in the subtraction of 0.05 eV, to account for the difference between zero point and the first vibronic level, was considered. For the resulting optimized geometries time dependent DFT calculations (using the same functional and basis set as those in the previous calculations) were performed to predict the vertical electronic excitation energies.

## 3 Results and discussion

### 3.1 Intercalation of the ketodiacid 3 in a Zn-Al layered double hydroxide

**FIGURE 1:**
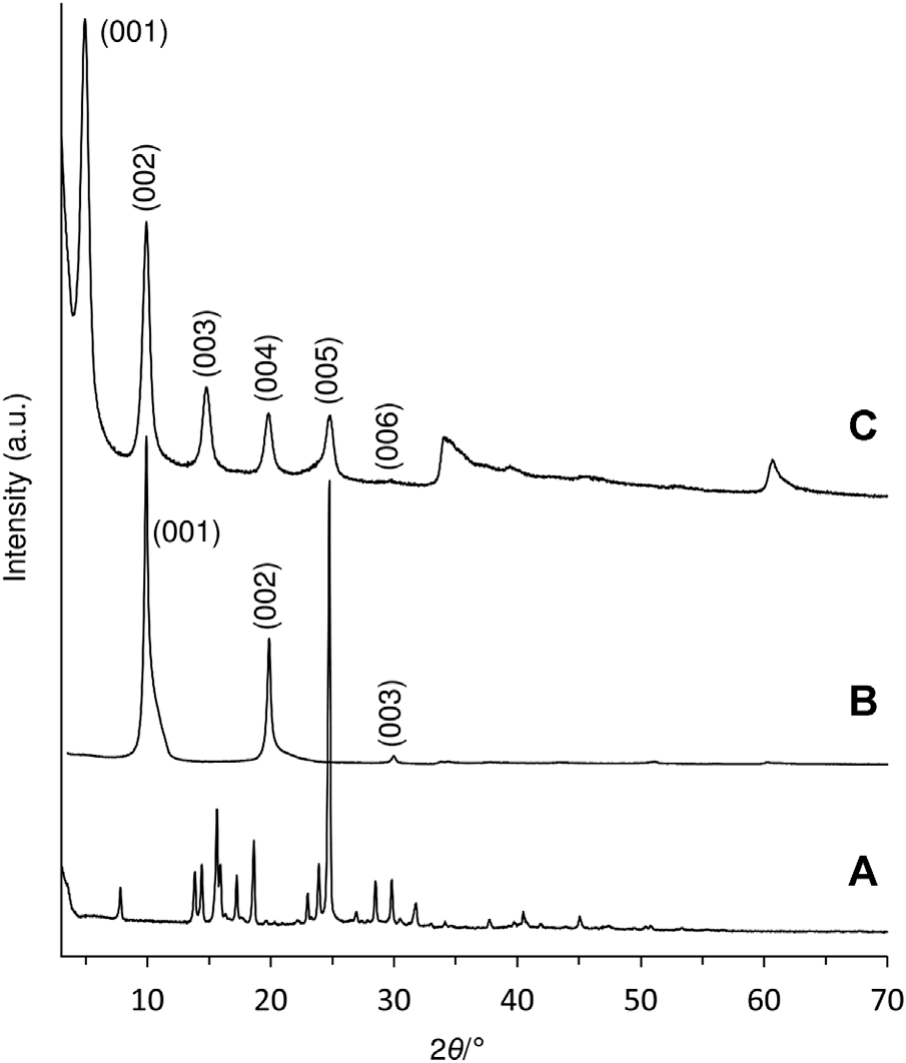
PXRD patterns of **(A)** ketodiacid **3**, **(B)** LDH-NO_3_, and **(C)** LDH-KDA3.

A Zn-Al LDH intercalated by the deprotonated form of the diketoacid **3** was prepared by a direct coprecipitation method. The PXRD pattern of the resultant solid, designated as LDH-KDA3, is typical of LDHs containing organic guests (Figure 1C). Five equally spaced basal (00*l*) reflections are observed at 2*θ* angles below 30°. The sharpness of the basal reflections indicates that the layer structure is well-ordered, i.e., the interlayer spacing is highly regular. On the other hand, the presence of several weak, broad and asymmetric non-basal reflections at 2*θ* angles above 30° indicates disorder in the layer stacking, such as a turbostratic distortion or an intergrowth of the rhombohedral and hexagonal polytypes (Bellotto et al., 1996; Evans and Slade, 2006). Since the type of polytype cannot be determined with any degree of certainty, the basal reflections have been indexed as a one-layer polytype (001, 002, 003, etc.). The interlayer spacing (*c*_0_) can be calculated from averaging the positions of the five 00*l* harmonics: *c*_0_ = (*d*_001_ + 2*d*_002_ + 3*d*_003_ + 4*d*_004_ + 5*d*_005_)/5 = 18.0 Å. This is almost exactly double the value of *c*_0_ (8.89 Å) for the reference material LDH-NO_3_ (Figure 1B).

**FIGURE 2:**
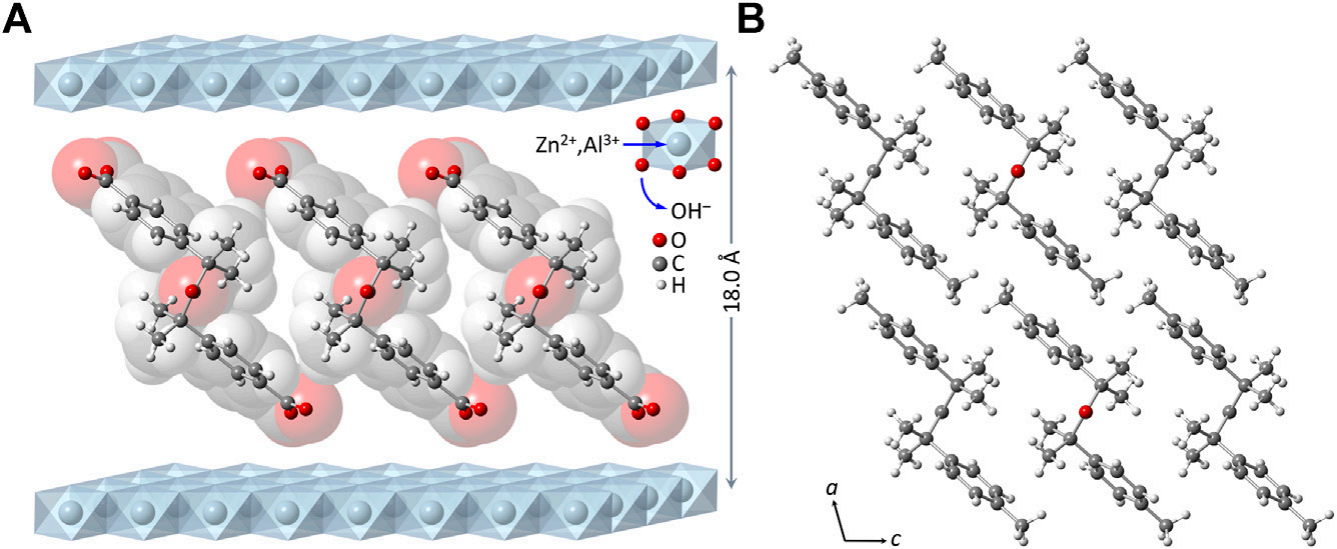
**(A)** Structural model (ball-and-stick diagram superimposed on a spacefilling, van der Waals-based representation) for the monolayer arrangement of 4,4′-(2,4-dimethyl-3-oxopentane-2,4-diyl)dibenzoate anions in the material LDH-KDA3. **(B)** View of the crystal packing of **2** down the crystallographic *b* axis.

Subtracting the hydroxide layer thickness of 4.8 Å from the interlayer spacing of 18.0 Å for LDH-KDA3 gives a gallery height of 13.2 Å. To the best of our knowledge, no crystal structure has been reported for the ketodiacid **3**. A structure has, however, been deposited with the Cambridge Structural Database (Groom et al., 2016) for the synthetic precursor to **3**, i.e., 2,4-dimethyl-2,4-di-*p*-tolylpentan-3-one (**2**) (CSD Refcode WIVHOZ). A realistic model for the molecular structure of the deprotonated form of **3** was created by extracting a molecule of **2** from the crystal structure and replacing the *p*-tolyl methyl groups by carboxylate groups with a fixed C‒O bond length of 1.25 Å and a <OCO angle of 125°. Figure 2 shows a schematic representation of a guest orientation that is calculated to give the experimentally observed gallery height of 13.2 Å. In this model, the guest molecules are markedly inclined from the normal to the hydroxide layers; the molecular axis defined by a vector joining the two carboxylate carbon atoms is at an angle of *ca*. 52° to the hydroxide surface of the layers. Apart from the fact that this arrangement gives a gallery height of 13.2 Å, there are other features that support this model: 1) hydrogen-bonding interactions between the carboxylate oxygen atoms and the layer hydroxyl groups are fortified, while positioning the hydrophobic dicumyl ketone section of the molecule towards the center of the interlayer region; 2) the inclined orientation may allow an efficient guest packing mode in which molecules can interlock as represented in Figure 2. The last argument is reinforced by the fact that a strikingly similar arrangement is present in the crystal packing of compound **2**, when viewed down the crystallographic *b* axis, as shown in Figure 2B.

**FIGURE 3:**
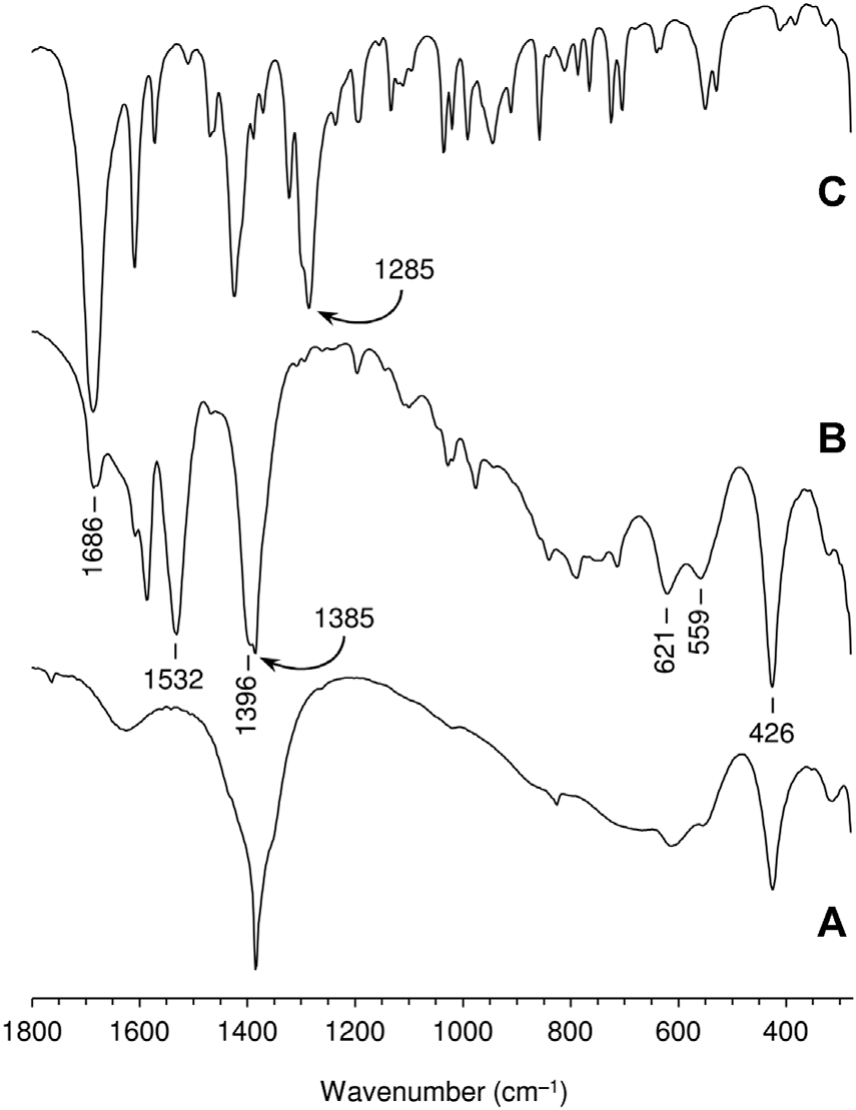
FT-IR spectra in the range 280–1800 cm^−1^ of **(A)** LDH-NO_3_, **(B)** LDH-KDA3, and **(C)** ketodiacid **3**. The frequencies of selected bands are indicated.

**FIGURE 4:**
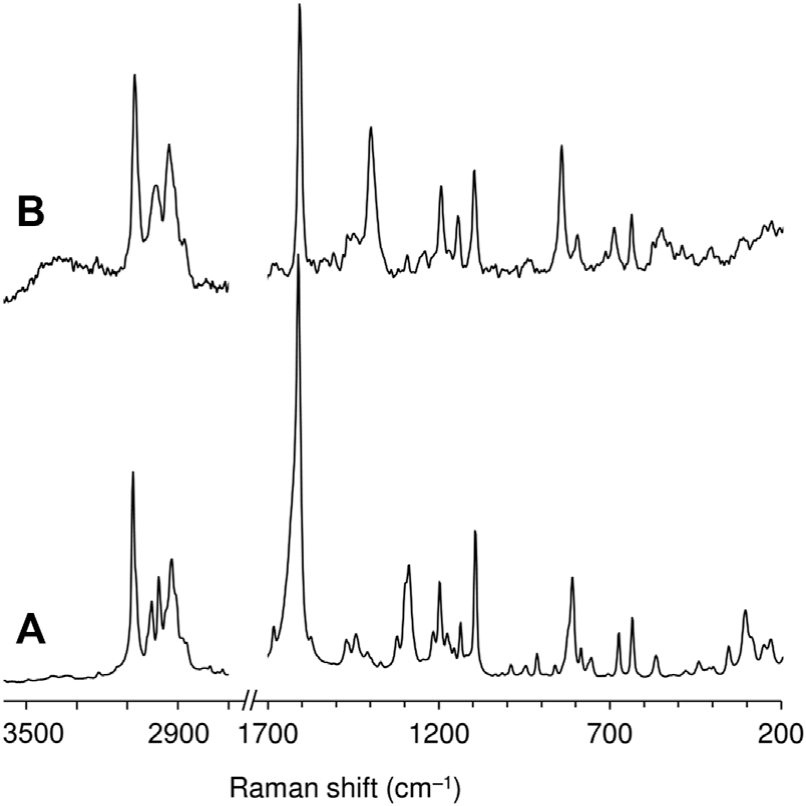
FT-Raman spectra in the ranges 200–1,700 and 2,700–3,600 cm^−1^ of **(A)** ketodiacid **3** and **(B)** LDH-KDA3.

The FT-IR spectra of the free ketodiacid **3** and the intercalated material LDH-KDA3 are shown in Figure 3. The spectrum of **3** is dominated by the very strong absorption at 1,686 cm^−1^ assigned to overlapping *ν*(C=O) bands of the ketone and carboxylic acid groups. LDH-KDA3 displays this band at the same frequency, albeit with reduced relative intensity, and it is therefore assigned to *ν*(C=O) of the ketone group. The deprotonation of the carboxylic acid groups of the guest molecules in LDH-KDA3 is confirmed by the appearance of two strong bands attributable to *ν*_sym_(CO_2_) (1,396 cm^−1^) and *ν*_asym_(CO_2_) (1,532 cm^−1^) vibrations, together with the absence of a *ν*(C‒O) band (present at 1,285 cm^−1^ for **3**). A weak, sharp band at 1,385 cm^−1^ that overlaps with the *ν*_sym_(CO_2_) band may be due to an asymmetric *ν*_3_ stretching mode of nitrate ions (present *via* cointercalation with **3** and/or formation of a secondary LDH-NO_3_ phase). Bands at 1,586 and 1,609 cm^−1^ for the intercalated LDH are assigned to aromatic ring stretching vibrations. Below 700 cm^−1^, the three main bands observed at 426, 559 and 621 cm^−1^ are attributed to the characteristic Zn/Al-OH lattice translation modes of Zn-Al LDHs (Kloprogge et al., 2004). Similar lattice mode bands are observed for the reference nitrate-form material LDH-NO_3_ (Figure 3A). The Raman spectrum of LDH-KDA3 is fully consistent with the FT-IR spectrum, showing a very strong band at 1,606 cm^−1^ (*ν*_ring_) and a strong band at 1,399 cm^−1^ [(*ν*_sym_(CO_2_)] (Figure 4).

**FIGURE 5:**
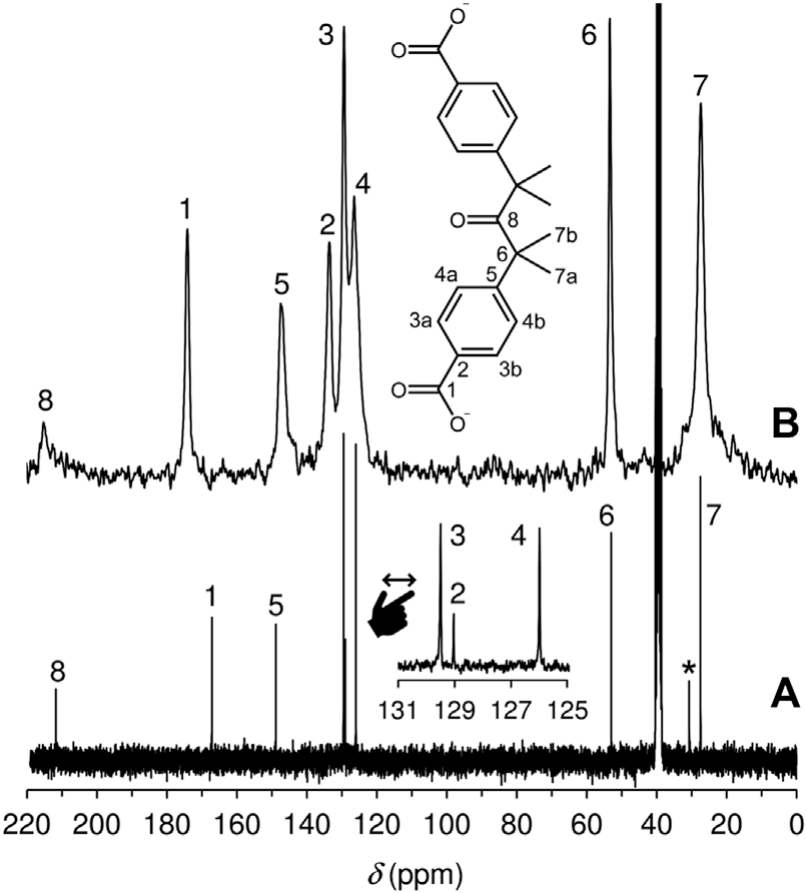
**(A)** Solution ^13^C{^1^H} NMR spectrum of the ketodiacid **3** in DMSO-d_6_. The resonance labelled with an asterisk is due to residual acetone. **(B)** ^13^C{^1^H} CP MAS NMR spectrum of LDH-KDA3.

The solid-state ^13^C{^1^H} CP MAS NMR spectrum of LDH-KDA3 is shown in Figure 5 alongside the solution spectrum of the ketodiacid **3** in DMSO-d_6_. The LDH displays eight distinct resonances that have a clear counterpart in the solution spectrum. Thus, the signals for the aliphatic (20–60 ppm), aromatic (120–150 ppm) and carbonyl (170–220 ppm) carbon atoms of LDH-KDA3 (and **3**, given in parentheses) are attributed as follows: (*δ*, ppm) CH_3_ at 27.4 (27.5), *C*(CH_3_)_2_ at 53.4 (53.0), phenylene-CH at 126.5, 129.4, 133.6, and 147.2 (126.0, 129.0, 129.5, 148.9), ‒CO_2_^−^ at 174.1 (167.1), and C=O at 215.1 (211.7). Hence, as expected, the most significant change is the downfield shift of the carboxylate resonance owing to deprotonation.

**FIGURE 6:**
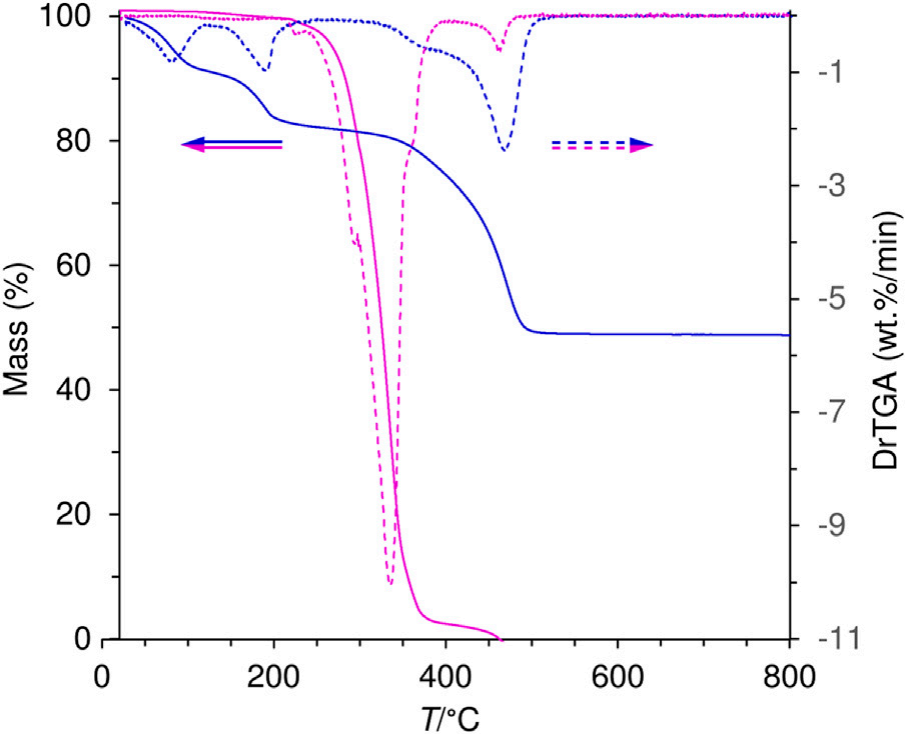
TGA (solid lines) and corresponding DTG (dashed lines) curves for the ketodiacid **3** (magenta) and LDH-KDA3 (blue).

TGA revealed that the organic guest anion in LDH-KDA3 starts to decompose at a significantly higher temperature than that determined for the free ketodiacid **3** (Figure 6). The intercalated LDH shows three main weight loss steps between ambient temperature and 500 °C corresponding to removal of physisorbed and cointercalated water molecules (8.9% mass loss up to 150°C), dehydroxylation of the hydroxide layers (6.9% loss in the range 150–210°C), and decomposition of the organic guest (31.2% loss in the range 330–500°C). The enhanced thermal stability of the intercalated ketodicarboxylate anions is underscored by the shift of the decomposition onset from about 230°C for **3** to 330°C for LDH-KDA3, as well as the shift of the maximum of the differential thermogravimetric (DTG) curve from 335°C for **3** to 470°C for LDH-KDA3. Comparable increases in thermal stability of organic carboxylates (vs. the initial free acid or sodium salt) have been reported for other systems, including Zn-Al LDHs containing intercalated benzenecarboxylate derivative anions, e.g., positive shifts of ca. 150°C for 5,5′-methylenedisalicylic acid (Cui et al., 2010), 130°C for aurintricarboxylic acid (Zhu et al., 2011), and 50°C for benzene carboxylate and 4-hydroxy-benzene carboxylate (Fleutot et al., 2012). These improvements in thermal stability are attributed to the formation of an ordered supramolecular structure with significant interactions (including electrostatic interaction between opposite charges, hydrogen bonding and van der Waals interactions) between the organic guest and the host layer.

**FIGURE 7:**
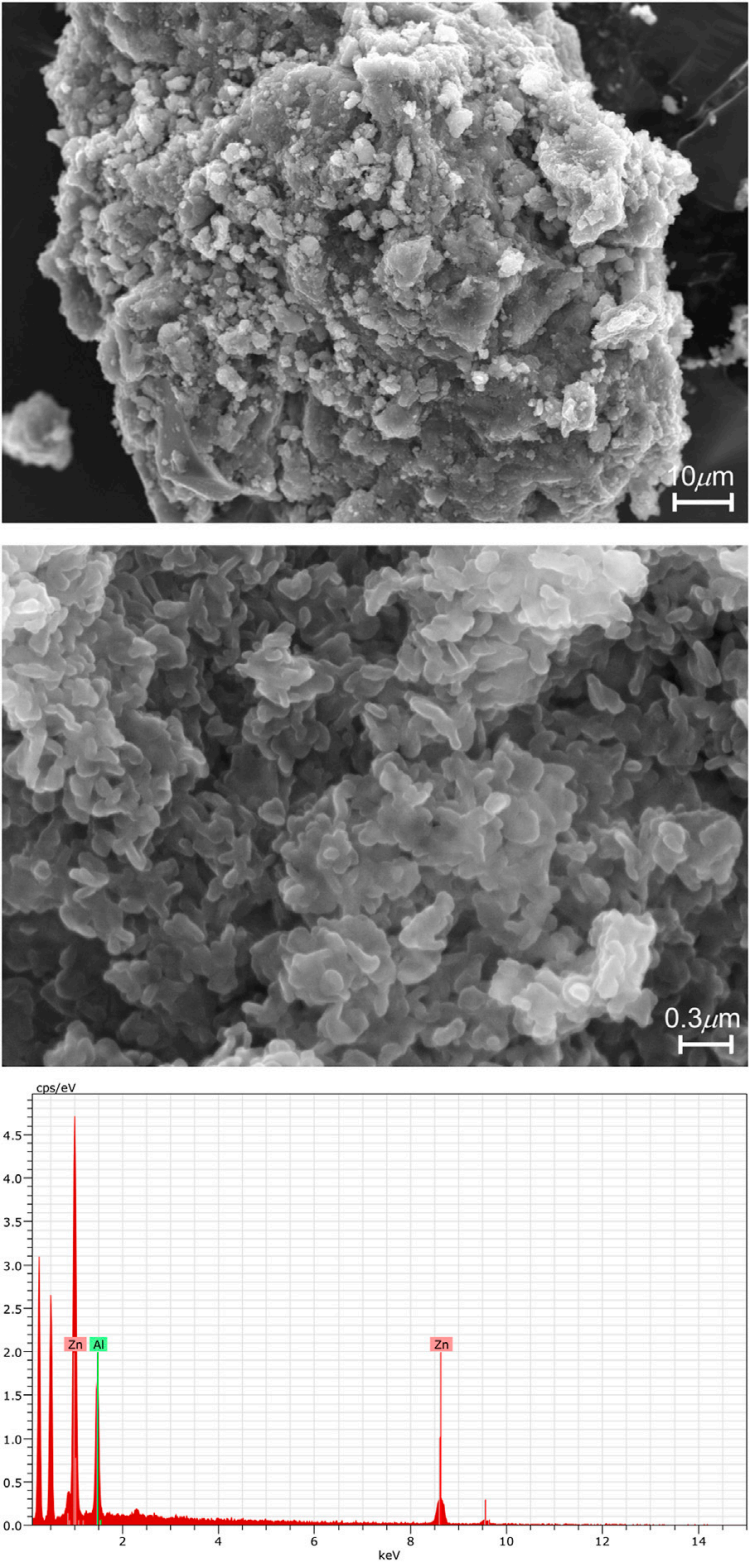
Representative SEM images under different magnifications and EDS spectrum of LDH-KDA3.

The phase purity of the material LDH-KDA3 was verified by SEM and EDS (Figure 7). The morphology of the intercalated LDH consisted of large aggregates of irregular sheet-like nanoparticles. EDS analyses indicated a uniform Zn/Al atomic ratio of 2.15 ± 0.05, which is quite close to the initial value of 2 in solution. The slight increase may have been caused by some leaching of aluminium during excessive washing with water. No evidence was found for secondary phases containing Zn (e.g., ZnO) and/or Al (e.g., Al_2_O_3_), in agreement with the PXRD data.

From CHN, EDS, and TGA analyses, together with the other characterization data, the formula Zn_4.3_Al_2_(OH)_12.6_(C_21_H_20_O_5_)_0.92_(NO_3_)_0.16_(H_2_O)_5_ is proposed for the material LDH-KDA3. As discussed above, the presence of a small amount of nitrate ions was indicated by FT-IR spectroscopy, and the nitrate content in the formula is consistent with microanalyses for N. Since neither the IR spectrum nor the solid-state ^13^C{^1^H} CP MAS NMR spectrum of LDH-KDA3 show peaks characteristic of carbonate ions, any interference from such species is deemed to be insignificant, and hence the presence of CO_3_^2−^ (in addition to NO_3_^−^) is not contemplated in the proposed formula.

To assess the stability of LDH-KDA3 in aqueous media, the material was incubated in two different biological buffers, 0.01 M PBS and 0.01 M HEPES, for 5 h at room temperature. No significant alterations in the IR spectrum or PXRD pattern of LDH-KDA3 were observed after these treatments (Supplementary Figures S2, S3), indicating that no structural changes took place, such as deintercalation of ketodicarboxylate anions by ion-exchange with phosphate ions in the PBS buffer solution.

### 3.2 Photolysis studies in the solid-state and in solution

#### 3.2.1 Characterization of photoproducts

**FIGURE 8:**
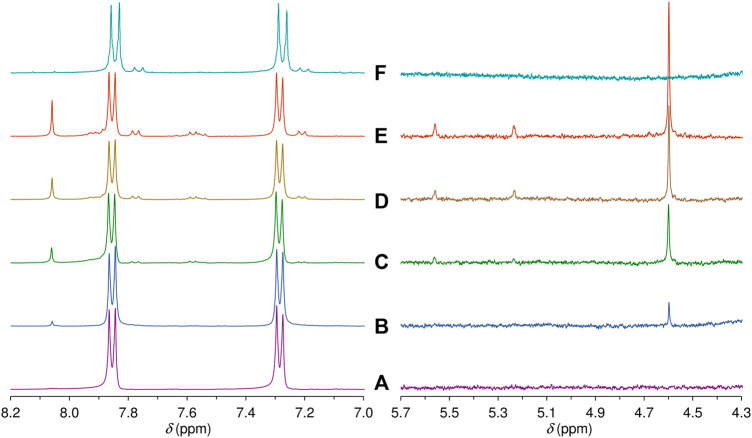
Aromatic and mid-field regions of the ^1^H NMR spectra obtained after irradiating a solution of the ketodiacid **3** in DMSO-d_6_ for **(A)** 0 h, **(B)** 3 h, **(C)** 5 h, **(D)** 8 h, and **(E)** 12 h. For comparison, **(F)** shows the same regions of the ^1^H NMR spectrum obtained in a DMSO-d_6_ solution of the irradiated solid i**3** (see main text for details).

**SCHEME 2:**
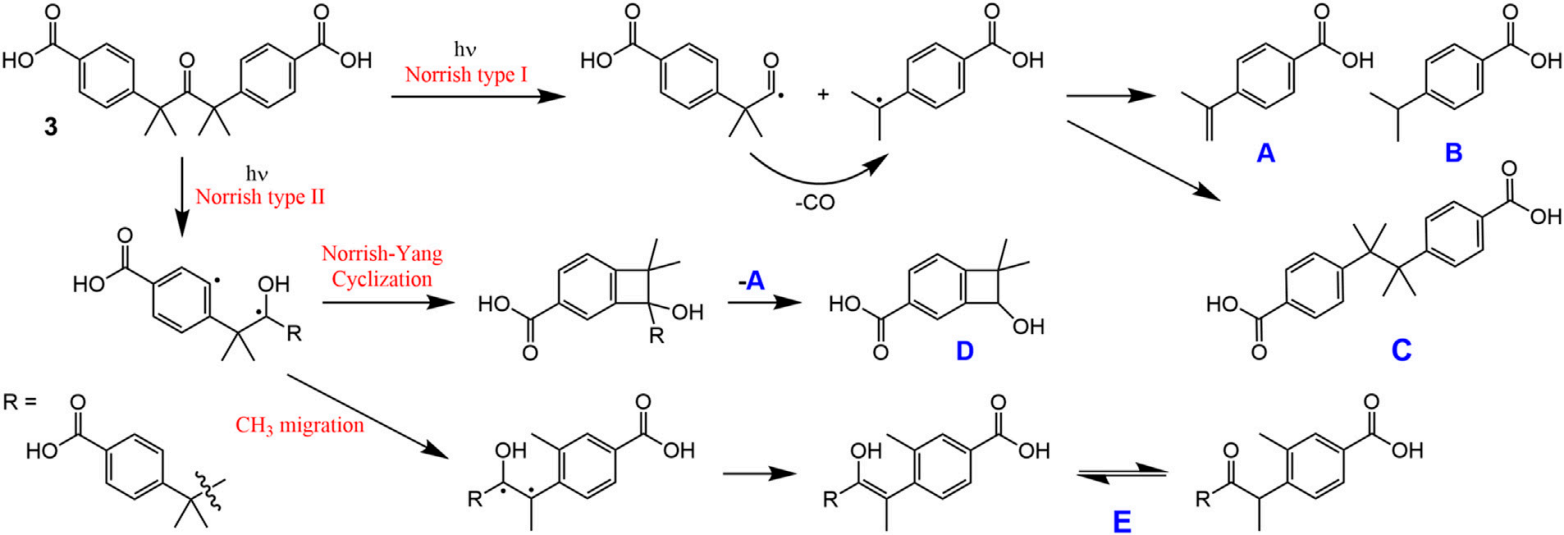
Possible photoproducts of **3** in solution.

Solid samples of **3** and LDH-KDA3 were irradiated with UV light for 12 h at room temperature, giving materials designated as i**3** and iLDH. The ^1^H NMR spectrum of i**3** in DMSO-d_6_ was recorded and compared with that for **3** (Figure 8, spectra A and F). In the region containing the resonances for the phenyl group hydrogens, **3** displays a pair of doublets at 7.28 and 7.85 ppm. For the irradiated sample i**3**, an additional pair of weak doublets is observed at 7.20 and 7.76 ppm, which are attributed to the diphenylethane combination product of photodecarbonylation (structure **C** in Scheme 2) (Zhang et al., 2007; Family and Garcia-Garibay, 2009). Signals due to other photoproducts, e.g., disproportionation products **A** and **B** in Scheme 2 (Family and Garcia-Garibay, 2009), were not observed. The high chemoselectivity of the photodecarbonylation reaction in the solid-state agrees with results reported by Family and Garcia-Garibay (2009). On the other hand, the relatively low photoreactivity observed in our work does not tally with the results of Family and Garcia-Garibay (2009) who reported conversions of 33% and 100% after room-temperature photolysis of **3** for 1 h and 5–6 h (using a similar experimental setup). Differences in sample crystallinity and structure may account for the contrasting photoreactivities observed. Zhang et al. (2007) found that the photodecarbonylation of crystalline hexasubstituted ketones could be quenched by the formation of intermolecular H-bonds involving the carbonyl (C=O) group. Although the PXRD pattern of **3** (Figure 1A) matches quite well with that reported by Family and Garcia-Garibay (2009) in terms of peak positions and relative intensities, the crystallinity of the sample obtained in the present work may be higher, which could imply differences in the long-range crystal packing and H-bonding networks.

The FT-IR spectrum of the irradiated solid iLDH was identical to that of the parent material LDH-KDA3 (not shown here). There was no detectable alteration in the relative intensity of the band assigned to *ν*(C=O) of the ketone group, suggesting that very little or no photodecarbonylation had occurred. This was investigated further by performing deintercalation (DI) reactions in which LDH-KDA3 and iLDH were incubated overnight in a solution of Na_2_CO_3_ at room temperature. The PXRD patterns and FT-IR spectra of the resultant solids (designated as LDH^DI^ and iLDH^DI^) confirmed that complete exchange of organic guest ions for carbonate ions had taken place to give Zn-Al-CO_3_ LDHs with the characteristic interlayer spacing of 7.6 Å (exemplified in Supplementary Figures S2D, S3D for iLDH^DI^). Second solids designated as KDA^DI^ and iKDA^DI^ were recovered from these experiments by evaporation of the exchange solutions to dryness. ^1^H NMR analysis of the phenyl group hydrogens of iKDA^DI^ confirmed, through comparison with the spectra for KDA^DI^ and the disodium salt of **3**, the presence of the unchanged ketodiacid (as the disodium salt), in agreement with the FT-IR spectrum of iLDH (Supplementary Figure S4).

To compare with the solid-state photolysis results, a solution of **3** in DMSO-d_6_ was irradiated at room temperature with UV light and ^1^H NMR spectra were recorded for different irradiation times (Figure 8). After an irradiation time of 3 h, new signals start to appear in the aromatic region, and these grow in relative intensity with further irradiation up to 12 h. The new doublets at 7.21 and 7.77 ppm are assigned to the diphenylethane combination product **C**. In contrast to the photolysis of **3** in the solid-state, the photolysis of **3** in solution is less selective, with additional signals being observed in the aromatic region, namely several weak resonances in the spectral ranges of 7.52–7.62 and 7.88–7.95 ppm, and a relatively intense singlet at 8.06 ppm. The weaker resonances could be due to **A** and/or **B** (Scheme 2). The ^1^H NMR spectrum of **B** in DMSO-d_6_ has been independently reported by two groups, and in both studies the compound gave doublets at 7.36–7.37 ppm and 7.86–7.88 ppm (Yang et al., 2014; Liu et al., 2018). Since no doublet appeared around 7.36 ppm upon photolysis of the solution of **3** in DMSO-d_6_, we exclude the formation of **B**, favoring instead the formation of **A**, which may be responsible for the weak doublet centered at 7.58 ppm and an additional signal in the 7.88–7.95 ppm range. These assignments are made by comparison with the spectrum reported for the methyl ester of **A** in CDCl_3_, which exhibited doublets at 7.51 and 8.01 ppm (Matsumoto et al., 1994). The postulation of **A** as a photoproduct is further supported by the appearance of weak singlets at 5.23 and 5.56 ppm [cf. 5.19 and 5.46 ppm for methyl 4-isopropenylbenzoate (Matsumoto et al., 1994)], assigned to the vinylidene hydrogens (Figure 8).

In addition to the signals around 5.5 ppm, the mid-field region of the ^1^H NMR spectra of the irradiated DMSO-d_6_ solution of **3** shows the growth of a singlet resonance at 4.60 ppm (Figure 8). This signal and the one at 8.06 ppm are not due to any of the Norrish type I (α-cleavage) photoproducts (**A**, **B**, and **C**) shown in Scheme 2. Hence, an alternative photoreaction sequence must be considered. One likelihood is the Norrish type II pathway (Oelgemöller and Hoffmann, 2016; Albini, 2021), which starts with the intramolecular abstraction of a γ-hydrogen to produce a 1,4-biradical (Scheme 2). In the case of **3**, the final products could include the dimethylbenzocyclobutenol derivative **D** (*via* Norrish-Yang cyclization) and the keto-enol system **E** (*via* a methyl migration reaction) (Scheme 2), which could both explain the new singlet observed in the aromatic region of the ^1^H NMR spectra.

**FIGURE 9:**
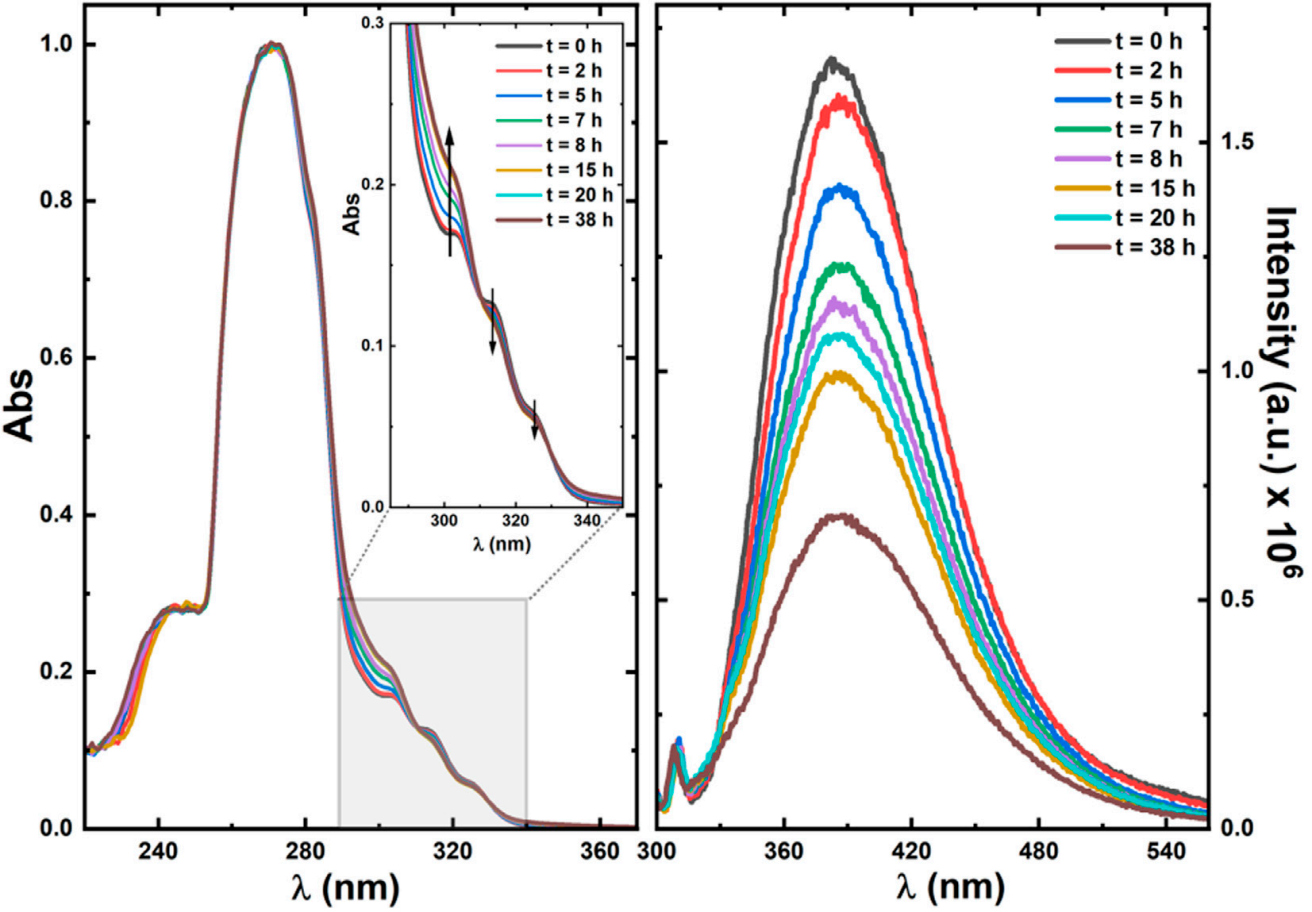
Normalized absorption (left) and fluorescence emission (right) spectra of ketodiacid **3** in DMSO-d_6_ solution with different irradiation times. In the absorption spectra the shape of the spectra for wavelength values below 260 nm mirrors the solvent’s cut-off, and the vertical arrows indicate the increase (∼304 nm) and decrease (∼314 nm and ∼327 nm) of the absorption bands.

To better understand the behavior of **3** under irradiation, a more comprehensive photophysical study, involving excitation of this compound in DMSO-d_6_ followed by UV-Vis and fluorescence spectroscopy, was undertaken. Figure 9 shows the absorption (left) and fluorescence (right) spectra upon irradiation for different periods up to a total time of 38 h (with the 158.5 W medium pressure Ultraviolet mercury arc lamp as described in Section 2.3).

The emission spectra were collected with excitation (*λ*_exc_) at 290 nm to avoid the superimposition of the Raman peak of the solvent. The dependence of the absorption spectra of ketoacid **3** on the irradiation time shows an increase of the 304 nm band and a decrease of the ∼314 nm and ∼327 nm bands. Although an isosbestic point could not be selected for excitation to obtain the emission spectra (to avoid the overlap of the Raman peak of the solvent in the emission spectra), with the *λ*_exc_ = 290 nm used for excitation (close to the peak maximum of 304 nm) the absorbance value increases with the irradiation time, whereas the emission band (with peak maxima, *λ*_em_, at 390 nm) decreases with the irradiation time. This shows that a new and less emissive compound is being formed at the expense of photodegradation of ketoacid **3** (see Figure 9).

Further rationalization of this behavior arrives with the predicted—from TDDFT calculations—absorption and emission maxima of ketoacid **3** and of the photoproducts (**A–E**). Indeed, for ketoacid **3** and photoproduct **E**, an absorption band (corresponding to an n,π* transition) with maxima at ca. 316–317 nm (predicted from TDDFT calculations) matches with the longest wavelength vibronically resolved absorption band (wavelength range 290–350 nm, see Supplementary Table S1 and Supplementary Figure S5). The other photoproducts (compounds **A**–**D**) do not show evidence of theoretically predicted low energy n,π* forbidden transitions. Moreover, the oscillator strength associated with the emission at 380 nm (Figure 9) of ketoacid **3** (*f* = 0.01) is higher than that of photoproduct **E** (*f* = 0.002), thus attesting, once more, that formation of this photoproduct is highly favorable and takes place through the proposed Norrish type mechanism (see Scheme 2; Supplementary Table S1).

Despite the above results, compound **D** is not excluded as a photoproduct formed from the photolysis of **3** in solution, since it appears to provide the best overall fit with the new NMR resonances, with the singlets at 4.60 and 8.06 ppm being assigned to the secondary alcohol methine proton and the isolated CH proton in the aromatic ring, respectively. The former assignment agrees with data recently reported for similar dimethylbenzocyclobutenols, differing only in the substituent on the benzene ring (F, Cl or Me instead of CO_2_H), which displayed the methine resonance in the range 4.7–4.8 ppm with CDCl_3_ as solvent (Chen et al., 2021). The remaining two aromatic protons of compound **D** may be responsible for the weak doublet centered at 7.55 ppm and an additional signal in the 7.88–7.95 ppm range. It is noteworthy that the proposed pathway leading to **D** also leads to the formation of **A**, which strengthens the assignment of the new resonances at 5.23, 5.56, 7.58, and 7.88–7.95 ppm to **A**.

#### 3.2.2 Myoglobin assays to measure photoinduced release of CO

The well-established Mb assay was used to assess the capabilities of ketodiacid **3** and LDH-KDA3 for the release of CO. The experiment, as previously described, relies on the spectrophotometric detection of the conversion of deoxy-Mb to MbCO (Motterlini et al., 2002).

**FIGURE 10:**
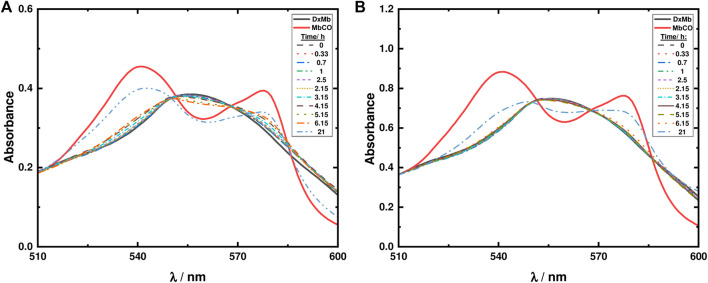
Mb assays for ketodiacid **3** (2.6 mM) using a deoxy-Mb (DxMb) concentration of approximately **(A)** 30 μM and **(B)** 60 μM.

With ketodiacid **3**, two different samples, with a final Mb concentration of approximately 30 and 60 μM, and a final ketodiacid concentration of 2.6 mM, were simultaneously irradiated at room temperature. Two sets of parallel assays were performed—one for 7 h (Supplementary Figure S6) and one for 21 h (Figure 10). Data in Figure 10 show the formation of MbCO, indicating photodecarbonylation of **3**. However, after 21 h of irradiation the maximum concentration of formed MbCO was found to be ca. 0.70% (0.07 equiv. MbCO based on **3**) in the two experiments with different Mb concentrations.

**FIGURE 11:**
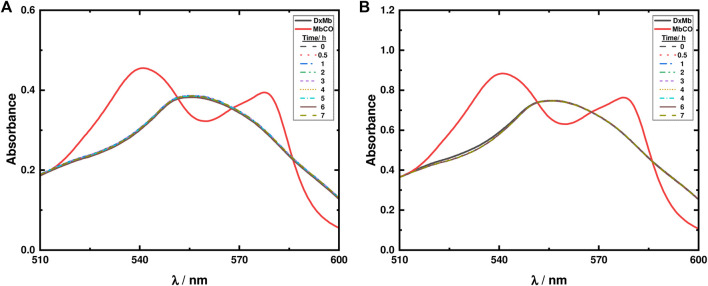
Mb assays for LDH-KDA3 (0.8 g/L) using a deoxy-Mb (DxMb) concentration of approximately **(A)** 30 μM and **(B)** 60 μM.

Regarding the material LDH-KDA3, suspensions of the solid in 0.1 M PBS buffer solution (0.8 g/L) were irradiated in the presence of two different concentrations of Mb (30 and 60 μM). As shown in Figure 11, after 7 h of irradiation no changes are observed in the absorption spectra, thus showing that in the LDH hybrid system, photodecarbonylation of ketodiacid **3** is inhibited.

Further analysis of the photodecarbonylation of **2**, the ditolyl precursor of **3**, was performed. Due to the low solubility of **2** in aqueous solution, the Mb assay was performed with a solution of 1% DMSO to solubilize **2** (Mansour and Shehab, 2018; Toscani et al., 2021). In this case, 5 mg of **2** were dissolved in 1 ml of DMSO, and an aliquot of 30 μl was taken to a final volume of 3 ml which contained a deoxy-Mb concentration of approximately 30 μM. Under these experimental conditions, release of CO was not detected as shown in Supplementary Figure S7. This may be a consequence of the low concentration (poor solubility) of compound **2**.

In comparison with results of photodecarbonylation obtained for other dicumyl ketones (Resendiz and Garcia-Garibay, 2005; Veerman et al., 2006), ketodiacid **3** has shown an extremely low percentage of photodecarbonylation in aqueous dispersion followed by the Mb assay, and LDH-KDA3 was not reactive at all. For the ketodiacid **3**, the competing Norrish type II pathway proposed above, involving conversion of the carbonyl group to a ketyl radical, may partly explain the low extent of the Norrish type I photodecarbonylation reaction and hence the low yield of MbCO in the Mb assay. An additional explanation is that the presence of carboxylic acid groups stabilizes, by intermolecular and/or (in the case of the LDH system) host-guest interactions, the compound, thus inhibiting triplet formation and α-cleavage to give an acyl-alkyl radical pair, followed by loss of CO from the acyl radical fragment. As mentioned above, other studies with crystalline dicumyl ketone derivatives are consistent with this observation since they showed that intermolecular hydrogen bonds can quench photodecarbonylation (Zhang et al., 2007).

## 4 Conclusion

In the present work we have demonstrated the enhanced thermal and photo-stability of a para-substituted dicumyl ketone intercalated in a layered double hydroxide. Due to the diminished photoreactivity, the study of the photochemistry of the ketodiacid (with respect to photoproduct selectivities) was limited to a comparison of results obtained for the compound in solution and in the crystalline state. The work has, nevertheless, worked as a proof of concept concerning the use of LDHs as hosts for the supramolecular organization of hexasubstituted ketones. Concerning the photoreactivity of **3** in solution, from photophysical and NMR data, further rationalized by TDDFT calculations, the formation of a less emissive photoproduct (keto-enol system **E**) *via* a methyl migration reaction is proposed, although the co-formation of photoproduct **D** (through Norrish-Yang cyclization) cannot be discarded. In future work it would be desirable to study more photoreactive ketones to probe the influence of the supramolecular ordering on the photochemistry of the guest molecules.

## Data Availability

The original contributions presented in the study are included in the article/Supplementary Material, further inquiries can be directed to the corresponding authors.
