# Supplementary material for: Enhanced thermal and photo-stability of a para-substituted dicumyl ketone intercalated in a layered double hydroxide
Source: Front Chem. 2022 Oct 10;10:1004586. doi: 10.3389/fchem.2022.1004586 (PMC9588950; doi:10.3389/fchem.2022.1004586)
Supplement: Supplementary file 1 [file DataSheet1.DOCX]

Supplementary Material


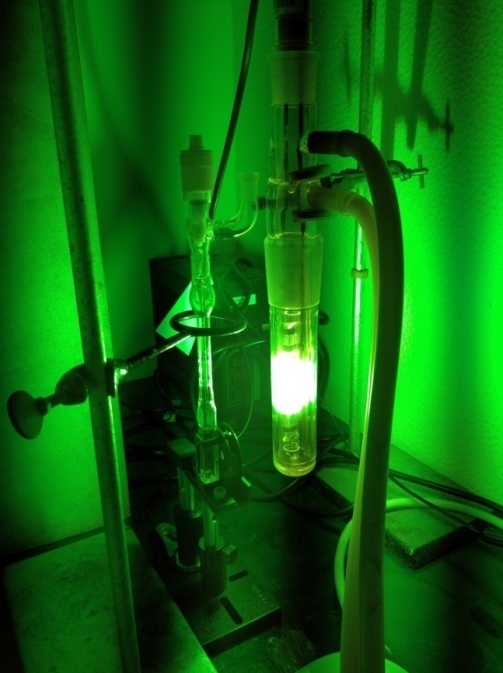


**FIGURE S1** Experimental arrangement for irradiation of solutions in the Mb assay.





**FIGURE S2** FT-IR spectra of LDH-KDA3 (a) and solids obtained after treatment of as-prepared or irradiated LDH-KDA3 in (b) 0.01 M HEPES (LDH^HEPES^), (c) 0.01 M PBS (LDH^PBS^), and (d) 0.17 M Na_2_CO_3_ (iLDH^DI^). Please refer to the main text for full details.





**FIGURE S3** PXRD patterns of LDH-KDA3 (a) and solids obtained after treatment of as-prepared or irradiated LDH-KDA3 in (b) 0.01 M HEPES (LDH^HEPES^), (c) 0.01 M PBS (LDH^PBS^), and (d) 0.17 M Na_2_CO_3_ (iLDH^DI^). Please refer to the main text for full details.

**FIGURE S4** ^1^H NMR of the phenyl group hydrogens in d_6_-DMSO solution of (a) sodium salt of ketodiacid **3**, (b) iKDA^DI^, and (c) KDA^DI^.

**TABLE S1** Absorption maximum theoretical (Calc.) values, in DMSO, for ketodiacid 3. The oscillator strength (*f*) and the respective electronic transition (Trans.) are described. Calculated transition energies for the emission of the optimized lowest singlet excited state (S_1_) are also reported. The two columns with the Calc. Trans. report the two lowest predicted electronic transitions (S_0_→S_1_ and S_0_→S_2_).

| **Compound** | **Calc. (*f*)** | **Trans.** | **Calc. (*f*)^a^** | **Trans.** | S_1_(*f*) |
| --- | --- | --- | --- | --- | --- |
| **3** | 249 (0.209) | H→L  ILCT  π→π* | 317 (0.0067) | H→L+1  ILCT  n→π* | 406 (0.010) |
| **A** | 271 (0.259) |  | N.O |  | 322 (0.759) |
| **B** | 230 (0.392) |  | N.O |  | 348 (0.001) |
| **C** | 235 (0.446) |  | N.O |  | 349 (0.001) |
| **D** | 224 (0.249) |  | N.O |  | 349 (0.002) |
| **E** | 247 (0.221) |  | 316 (0.0034) |  | 413 (0.002) |

^a^ N.O.: not observed by TDDFT quantum electronic calculations.


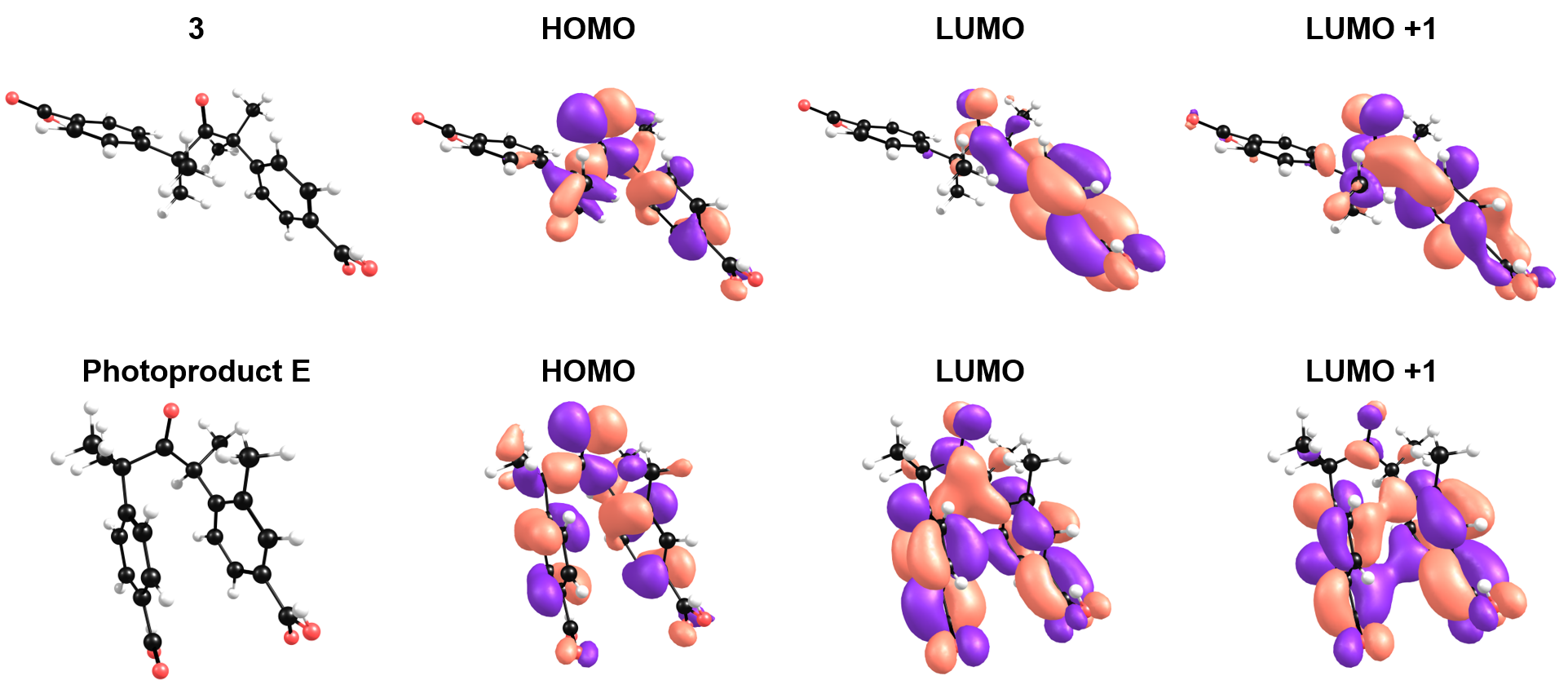


FIGURE S5 Representative molecular orbital contours for ketodiacid 3 and photoproduct E.


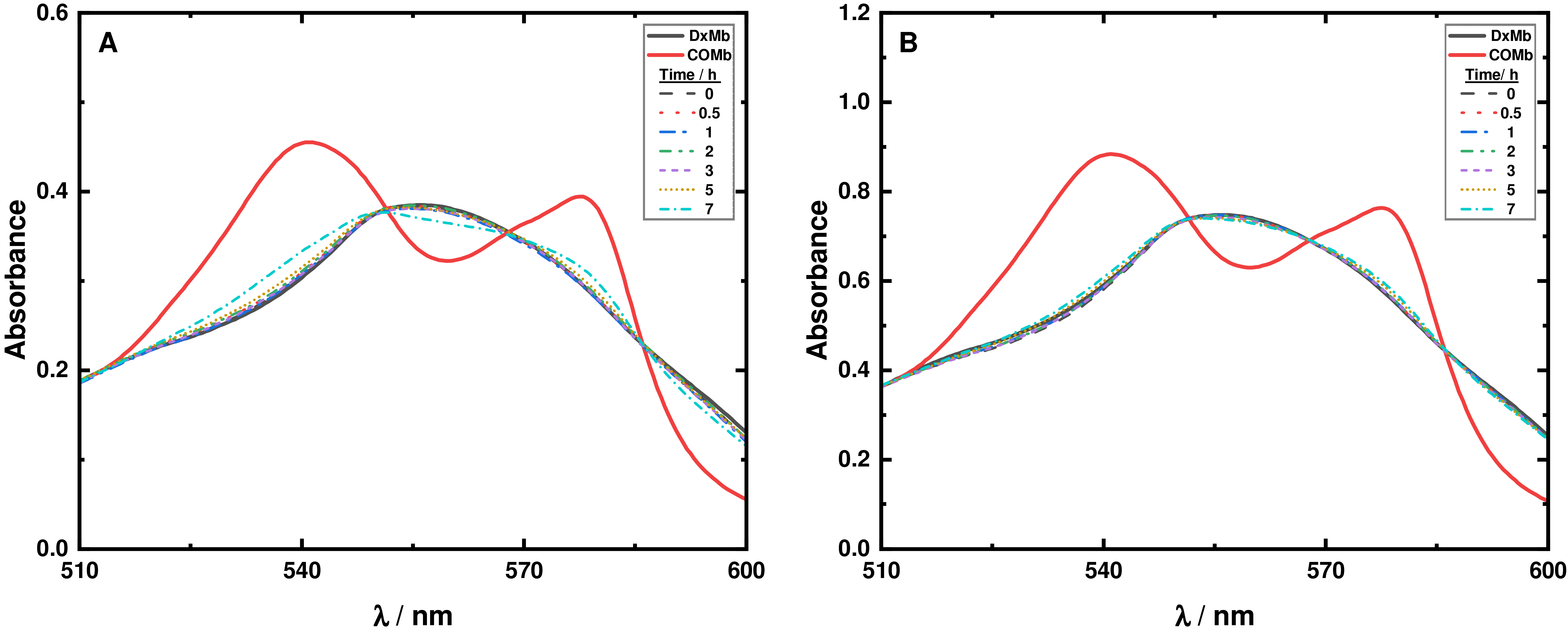


**FIGURE S6** Mb assays for ketodiacid **3** (2.64 mM) using a deoxy-Mb concentration of approximately (A) 30 μM and (B) 60 μM.


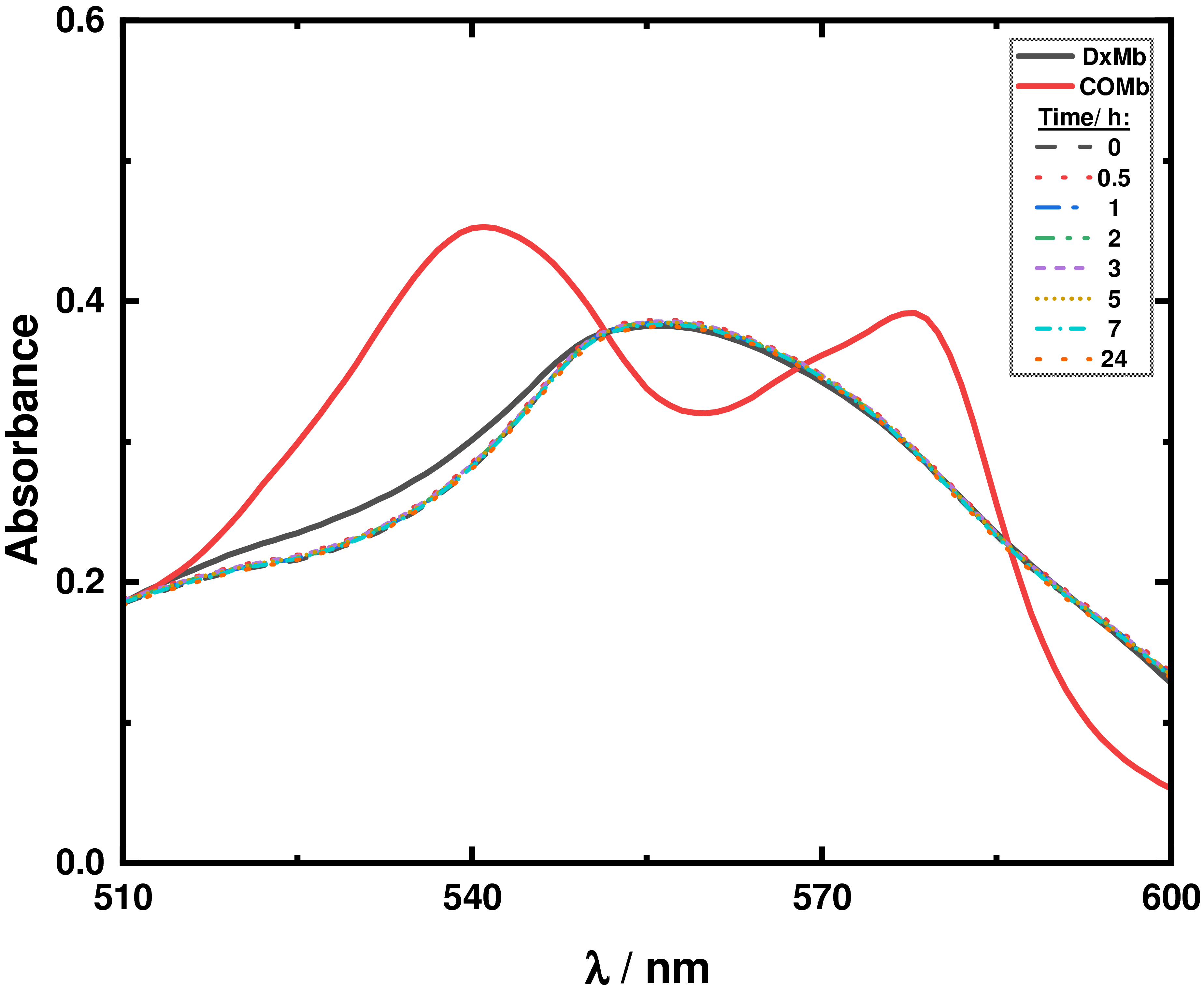


**FIGURE S7** Mb assays for **2** in a solution of 1% DMSO with [deoxy-Mb] = 30 μM.
